# The Use of Pupillometry in Autobiographical Implicit Association Test

**DOI:** 10.3389/fpsyg.2021.729897

**Published:** 2021-09-29

**Authors:** Tokihiro Ogawa, Natsu Todoriki, Michiko Tsuneoka

**Affiliations:** ^1^National Institute of Police Science, Chiba, Japan; ^2^Forensic Science Laboratory of Chiba Prefecture Police Head Quarter, Chiba, Japan

**Keywords:** autobiographical implicit association test, reaction time, pupil diameter, memory detection, forensic settings

## Abstract

The Autobiographical Implicit Association Test (aIAT) is a reaction time-based methodology to assess one's recognition of the truth value of propositions about an autobiographical episode. This study introduced pupillometry to examine its utility as an additional measure of aIAT. Participants blindly chose one of two cards and memorized it. They then underwent the aIAT to assess the cards they chose. The pupil diameter was larger in the block in which sentences related to the chosen card shared the same response key with sentences describing false events than the block in which sentences related to the chosen card shared the same response key with true-event sentences. Although preliminary, pupil measurement also yielded high efficiency in discriminating the chosen card. These results indicate that pupillometry can be used as a measure of aIAT.

## Introduction

The autobiographical Implicit Association Test (aIAT; Sartori et al., 2008) is a variant of the Implicit Association Test (IAT; Greenwald et al., 1998), which is applied to forensic settings such as crime investigations by law enforcement. It examines one's recognition of the truth value of propositions about an autobiographical episode (Verschuere et al., 2015). More specifically, the aIAT can be used to evaluate which of two alternative propositions (e.g., “I stole money from the cashbox” vs. “Someone else stole money from the cashbox”) one perceives as true. As in the standard IAT, the aIAT requires participants to engage in two types of categorization tasks. In one task, the participant has to provide a quick response to categorize sentences (e.g., “I'm in front of the computer”) according to their veracity. In the second task, the participant classifies sentences about two events (e.g., “I picked card number 4”) by their general topic, only one of which is true. In the combined blocks, these two tasks are mapped onto the same response keys. In a *compatible* block, true sentences share one of the two response keys with sentences describing the actual event. However, in an *incompatible* block, true sentences share the same response key with sentences about the fictional event. Sartori et al. (2008) reasoned that participants should respond faster in the compatible block than in the incompatible block. A review by Agosta and Sartori (2013) found that aIAT can identify true autobiographical events with over 90% accuracy. They argued that aIAT is a reliable test that can be easily and quickly implemented with inexpensive equipment and does not require special training. However, it should be noted that several factors, such as faking, use of negative sentences, and negative labels, could affect the accuracy of aIAT (Verschuere et al., 2009, 2015; Agosta et al., 2011).

The present study examined the efficacy of pupillometry as a measure of aIAT. Small fluctuations in pupil diameter are known to be linked to several aspects of cognitive and attentional processes, such as intensity of processing (Just and Carpenter, 1993) and processing load (Beatty, 1982; Beatty and Lucero-Wagoner, 2000). In addition, several studies have reported that pupil dilation is associated with reaction time (RT) costs in RT experiments, such as the Simon task (van Steenbergen and Band, 2013) and Stroop task (Laeng et al., 2011). Such covariations in pupil diameter and RT lead to the prediction that pupil diameter in IAT should be larger during the incompatible block than compatible blocks. If that is the case, pupil diameter would work as an additional non-invasive measure of the IAT. Indeed, the IAT has suitable features for pupil measurements. It manipulates only key assignment, while a presented stimulus set is identical across blocks, which allows for the control of confounding factors on pupillary responses such as luminance levels of visual stimuli. Furthermore, analyzing RT combined with a pupillary measure may provide clues to detect participants' attempts to fake in the IAT, which will be discussed later in detail. Thus, the use of pupillometry in the IAT may increase the availability of this method, especially in the aIAT, where faking is one of the obstacles to its utility (Verschuere et al., 2015). However, to the best of our knowledge, little is known about pupillary changes during IAT, even under a no-faking condition.

As the first step to examine the availability of pupillary measure in the IAT, the present study measured pupil diameter in a playing card aIAT similar to Experiment 1 in Sartori et al. (2008). Participants blindly chose one of two playing cards, the four of diamonds or the seven of clubs, and underwent the aIAT. To the best of our knowledge, this is the first attempt to introduce pupillometry into an IAT experiment.

## Method

### Participants

The participants included 43 adults (23 males and 20 females; mean age = 32.93 and SD = 4.89 years) with normal vision. This sample was employed to enable detection of a within-subject difference with a medium effect size (*d* = 0.5) and with a power of 0.8 in a two-tailed test while allowing some data loss. The study was approved by the research ethics committee of the National Research Institute of Police Science. All participants provided written informed consent and received 7,000 Rakuten Super Points (equivalent to approximately USD 64) for participation^1^.

### Materials and Apparatus

The stimuli were 20 short Japanese sentences (see Table 1), five for each stimulus category constructed in accordance with Experiment 1 in Sartori et al. (2008). We used ten Japanese sentences describing card choice, five for the four of diamonds, and the remainder for the seven of clubs. Five true and five false sentences were created. True sentences stated that the participant was engaged in a psychological experiment. False sentences described the participant engaging a study session held in the library.

**Table 1:** Sentences used in the autobiographical implicit association test.

**Category**	**Sentence** ^*^	**Ground truth**
True statement	I am in the Research institute	True for all participants
	I am in a little room	
	I am taking part in an experiment	
	I am in front of the monitor	
	I am sitting	
False statement	I am in a library	False for all participants
	I am in a large room	
	I am taking part in a study meeting	
	I am in front of a library shelf	
	I am standing	
4 of diamonds	I picked card number 4	True for the card 4 group; false for the card 7 group
	I put the card “four” in the envelope	
	I saw the 4 of diamonds	
	I put the 4 of diamonds in an envelope	
	I have the 4 of diamonds	
7 of clubs	I picked card number 7	True for the card 7 group; false for the card 4 group
	I put the card “seven” in the envelope	
	I saw the 7 of clubs	
	I put the 4 of diamonds in the envelope	
	I have the 7 of clubs	

* *Sentences were presented in Japanese in the experiment*.

All sentences were presented in white letters against a gray background (RGB-code: 128, 128, 128) on a 1920 × 1080 pixel LCD monitor placed at a distance of ~90 cm from the participant. Presentation was controlled by SuperLab 5.0.5 (Cedrus Corp.), which also recorded reaction times with millisecond accuracy using a response pad (RB-740, Cedurs Corp.) and sent signals indicating the start and end of the experimental blocks to the pupillometry device. The binocular pupil diameters were recorded at 60 Hz using an EMR ACTUS (Nac Image Technology Inc.).

### Procedure

The IAT session commenced immediately after the card was blindly selected. The IAT consists of seven blocks and took ~7 min on average from the start of the first block to the end of the 7th block including inter-block short break and time for instructions. In Block 1 (20 trials), participants classified sentences according to their categories by pressing the leftmost key in response to the four of diamonds sentences and the rightmost key for the seven of clubs sentences. In Blocks 2 and 5 (20 trials each), participants judged whether the presented sentence belonged to the true (with the leftmost key) or false categories (with the rightmost key). This key assignment was reversed in Block 5. Blocks 3 (20 practice trials) and 4 (40 main trials) were combined blocks in which all sentences were presented, and participants categorized a presented sentence according to the type of playing card or true/false dimension under the same key assignment. Blocks 6 (20 practice trials) and 7 (40 main trials) were also combined, but with the reversed key assignment for the true and false sentences of Block 5. Thus, blocks 4 and 7 were compatible and incompatible blocks, respectively, for the four of diamonds card group and vice versa for the seven of clubs card group. Throughout each block, relevant category-label words were presented at the top of the display as reminders. All stimuli and reminders were presented within an area from 460 to 1460 pixels on the horizontal axis and 0 to 640 pixels on the vertical axis, with the upper left as the origin. The participants were asked to respond as quickly and accurately as possible. Stimuli were presented with an inter-trial interval of 150 ms and remained until the participant responded. The computer recorded the reaction time from the onset of the stimulus to the response. A warning message was presented for 1,000 ms as feedback for an incorrect response. After all blocks had been completed, participants rated relative task difficulty of the blocks. These results were not analyzed and reported here.

### Pupil Data Acquisition and Preparation

Pupil data were converted to text files using EMR-dStream and then analyzed using a custom-made macro. Artifacts including blinks, recording errors identified by the eye recorder, and fixations outside the presentation area were corrected using linear interpolation. The mean pupil diameter from the right eye was calculated for the compatible and incompatible blocks. Since the duration of the main blocks differed from participant to participant depending on overall RTs (from 25 to 89 s; mean = 45.73± 10.25 s), mean pupil diameters of the first 25 s in the main blocks, labeled as the adjusted mean pupil, were also analyzed.

The mean RT for the compatible and incompatible blocks was also calculated in accordance with Greenwald et al. (1998). After discarding the first two trials of the main blocks, RTs outside of <300 ms and more than 3,000 ms were replaced with those boundary values. Error latencies were included in analyses. All RT values were log-transformed and averaged within a block.

The effect of compatibility (compatible vs. incompatible) on both pupil and RT measures was analyzed using paired *t*-tests. Statistical significance was set at *P* < 0.05. The effect size was expressed as Cohen's *d*.

## Results

Four participants were removed from analyses of pupil measures. They were discarded due to missing data points for more than 90% of analysis epochs (2 participants), postural change during the task (one participant), and exceptionally low correlation between right-left pupil diameter (one participant with *r* = 0.51) compared with other participants for whom correlations were higher than 0.80 with a median of 0.95. Two participants were missing their RT data due to a technical mistake. And two participants were discarded from RT analyses due to relatively frequent error responses (more than 16% of errors compared with the average error rate of 2.32 ± 3.91%). We collected binocular pupil diameters, RTs during the IAT blocks and self-reported difficulty of the combined blocks. But the left pupil diameter and the self-report measure are not reported here^2^. Thirty five participants were available for both pupil measurement and RT analyses.

**Figure 1:**
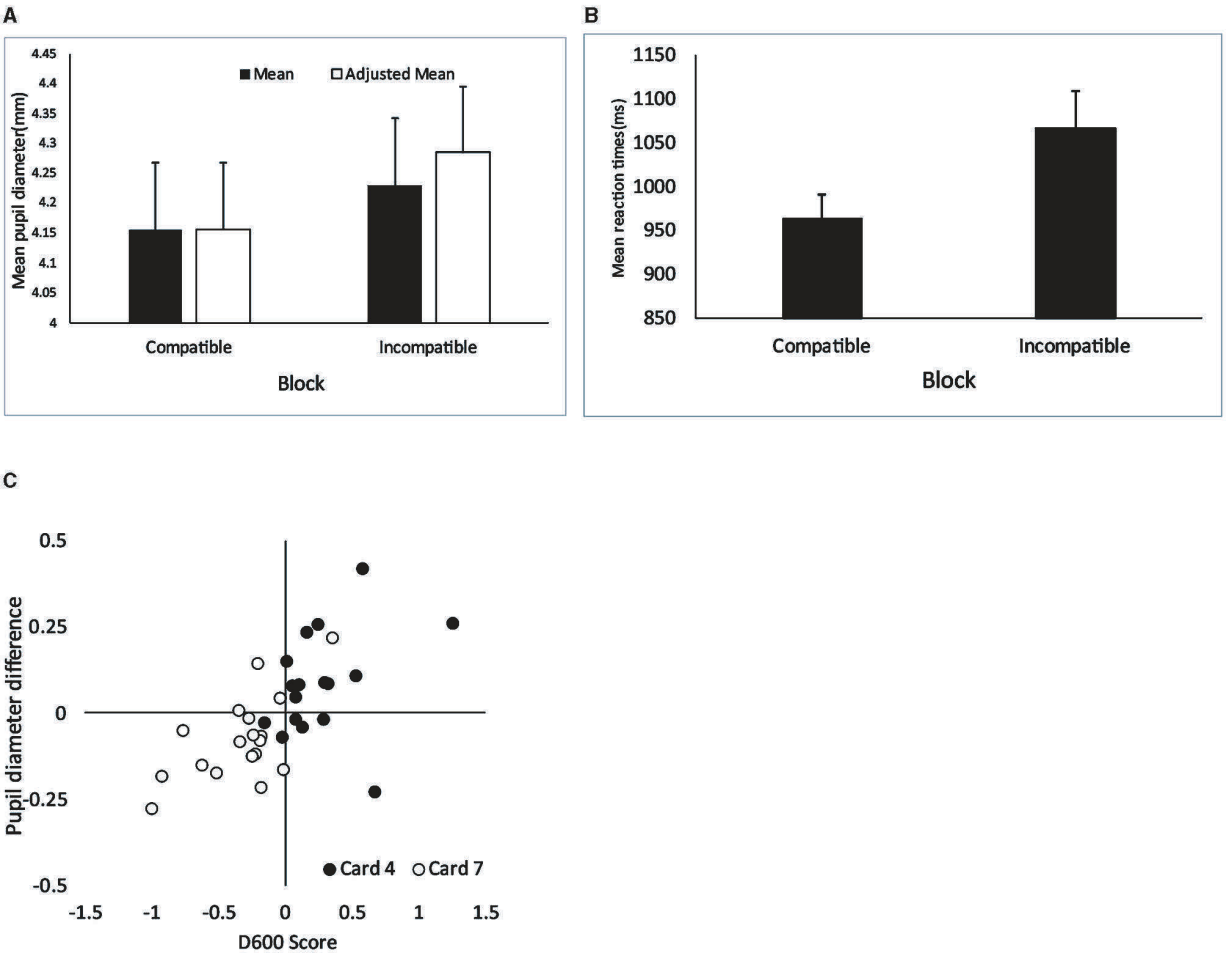
**(A)** Block mean pupil diameter and the adjusted block mean pupil diameter (means of initial 25 s in each block). **(B)** Block mean reaction time, which is rescaled from the log-transformed value. Standard errors based on the non-transformed, original value. **(C)** Relationship between pupil and behavioral measures. The pupil measure is the difference in mean pupil diameter (Block 7–Block 4). Bars indicate standard errors. Behavioral measure (reaction time) is expressed as the D_600_ score. Positive values imply smaller pupil diameter and faster response in Block 4 (the four of diamonds + true block) than in Block 7 (the seven of clubs + true block).

Paired *t*-tests indicated significant differences between compatible and incompatible conditions for the mean pupil [*t*_(38)_ = 3.55, *p* = 0.001, *d* = 0.57], adjusted mean pupil [*t*_(38)_ = 4.31, *p* < 0.001, *d* = 0.69], and RT [*t*_(38)_ = 4.51, *p* < 0.001, *d* = 0.72]. As shown in Figure 1, pupil diameter was larger in the incompatible block than in the compatible block. RT was slower for the incompatible block than for the compatible block, replicating the IAT effect.

Next, we assessed the between-group discrimination efficiency (the 4 of diamonds group vs. the 7 of clubs group) using area under receiver operating characteristic curves (AUCs). A difference measure was calculated by subtracting the mean pupil diameter in Block 4 from that in Block 7 so that positive scores indicated a stronger tendency to associate picking the four of diamonds with truth. For RT, we calculated the D600 score, also referred as D4 in Greenwald et al. (2003) that based on the difference in performance between the two types of combined blocks scaled by the standard deviation of the RT. D600 score is a specific type of D score where RT associated with incorrect responses is replaced with the mean of that block plus a 600-ms penalty. In the present study, a larger positive score is taken as an indication of the association between the four of diamonds and the truth. The groups differed in these differentiation indices [mean pupil diameter: *t*_(33)_ = 3.38, *p* = 0.002, *d* = 1.14; the adjusted mean pupil diameter: *t*_(33)_ = 4.23, *p* < 0.001, *d* = 1.43; and D600 score: *t*_(33)_ = 5.33, *p* < 0.001, *d* = 1.80]. The AUC was also significant with respect to the mean pupil diameter (0.810, 95% CI = 0.660–0.961), adjusted mean pupil diameter (0.859, 95% CI = 0.732–0.987), and D600 score (0.948, 95% CI = 0.863–1.00). AUCs in the D600 score tended to be larger than the mean pupil diameter (*Z* = 1.92, *p* = 0.054) but did not differ from the adjusted mean pupil diameter (*Z* = 1.52, *p* = 0.128). The D600 score positively correlated with pupil difference measures (*r* = 0.633, *p* < 0.001 for the mean pupil diameter; *r* = 0.691, *p* < 0.001 for the adjusted mean pupil diameter; see also Figure 1).

## Discussion

The present study conducted an aIAT experiment to examine the utility of pupillometry in the IAT. Since pupil diameter is sensitive to cognitive effort, we predicted that pupil diameter was larger in the incompatible block than in the compatible block, mirroring the conventional RT-based IAT. The results supported this prediction. In addition, pupillary measures yielded slightly less efficiency than RT, but still provided an excellent discrimination of groups according to a classification schema (AUC >0.800, Hosmer et al., 2013). These results were almost unchanged when the analyses used only initial pupillary data (i.e., the first 25 s of main blocks). However, the effect sizes and discrimination performance were numerically larger in the pupil measures of this restricted period. Overall, these results suggest that pupil diameter can serve as an additional measure of IAT.

Furthermore, we speculate that concurrent recording of pupil diameter can increase the availability of IAT. For example, one possible direction would be to explore the ability of pupillometry to detect faking in the IAT. Previous studies have shown that test outcomes can be altered by deliberately slowing responses in compatible blocks and/or speeding-up responses in incompatible blocks (Fiedler and Bluemke, 2005; Verschuere et al., 2009; Agosta et al., 2011; Hu et al., 2012). If deliberate slowing is associated with lower cognitive load due to the reduced effort to respond quickly, then RT and pupillometry are expected to produce opposing outcomes with longer RTs and smaller pupil diameters. Similarly, deliberate speeding-up is expected to be associated with faster RT and larger pupil diameter. If so, such paradoxical outcomes could be considered an indication of faking. Therefore, pupil measures may further improve the detection efficiency of fakers by combining the RT-based algorithm proposed by Agosta et al. (2011). Future studies should test if fakers and non-fakers can be discriminated by scrutinizing whether both measures produce compatible-incompatible differences in the same direction.

Several other points need to be further examined. The AUC results should be replicated and extended in future experiments with a larger sample size. Although there was no significant difference between the discrimination efficiencies between the pupil measures and the RT-based D600, the relative efficiency of pupil measures to the RT needs to be further clarified with a larger sample size. The present study used raw pupil diameter rather than stimulus-locked baseline-corrected pupillary changes. The results suggest that this measure works well in the IAT, but future studies should explore the optimal quantification of pupillary response (see also Attard-Johnson et al., 2019).

The present results show that pupillometry can be a useful new measure in the IAT. As noted at the outset, pupillometry is compatible with the methodological structure of the IAT, but its use has been scarce in the IAT literature, including aIAT literature. Possibly, since RT alone can produce relatively high performance in the aIAT, there has been little motivation to develop additional measures. A larger effect size and a higher AUC found for the RT measure suggest that this measure can contribute a lot to discrimination between Compatible and Incompatible conditions even if it is combined with pupillometry. In our view, RT measures would work well in most laboratory settings where participants make serious efforts to carry out experimental tasks. But in forensic settings, it may be difficult to expect people to be equally compliant with carrying out the task (Vrij, 2008). In these settings the use of multiple measures is expected to contribute not only to improving accuracy of the test (see also Hartwig and Bond, 2014), but also to discarding inappropriate cases. Moreover, consistent results from different channels can be taken as more convincing evidence. However, since the present study was based on a single experiment, further studies are needed to examine the utility of combining RT with pupillometry in different situations. We hope that the present study provides a springboard for the use of pupillometry in the aIAT study.

## Data Availability Statement

The raw data supporting the conclusions of this article will be made available by the authors, without undue reservation.

## Ethics Statement

The studies involving human participants were reviewed and approved by The internal ethical review board of the National Research Institute of Police Science. The patients/participants provided their written informed consent to participate in this study.

## Author Contributions

TO designed and performed the research and prepared the manuscript. NT contributed to the acquisition of the data and writing macros for data analyses. MT contributed to critical revision of the manuscript. All authors contributed to the article and approved the submitted version.

## Funding

This research was supported by the Japan Society for the Promotion of Science (JSPS), Grant-in-Aid for Scientific Research(C), Grant No. JP18K03195.

## Conflict of Interest

The authors declare that the research was conducted in the absence of any commercial or financial relationships that could be construed as a potential conflict of interest.

## Publisher's Note

All claims expressed in this article are solely those of the authors and do not necessarily represent those of their affiliated organizations, or those of the publisher, the editors and the reviewers. Any product that may be evaluated in this article, or claim that may be made by its manufacturer, is not guaranteed or endorsed by the publisher.

## Footnotes

^1^Participants took part in another experiment that had no relevance to the present study. The whole process took ~2 h. The payment also included transportation cost.

^2^Analyses of the left-eye data yielded almost identical results to the right eye data but somewhat reduced compatible-incompatible effect size (0.57 vs. 0.44 for the mean pupil and 0.69 vs. 0.58 for the adjusted mean pupil). We measured self-report difficulty for a preliminary purpose and decided not to report here.
